# Quality inspection of specific electronic boards by deep neural networks

**DOI:** 10.1038/s41598-023-47958-0

**Published:** 2023-11-24

**Authors:** Peter Klco, Dusan Koniar, Libor Hargas, Katarina Pociskova Dimova, Marek Chnapko

**Affiliations:** 1 Faculty of Electrical Engineering and Information Technologies, University of Zilina, Zilina, Slovakia; 2 Semikron-Danfoss, Vrbove, Slovakia

**Keywords:** Electrical and electronic engineering, Software

## Abstract

Reliability and lifetime of specific electronics boards depends on the quality of manufacturing process. Especially soldering splashes in some areas of PCB (printed circuit board) can cause change of selected electrical parameters. Nowadays, the manual inspection is massively replaced by specialized visual systems checking the presence of different defects. The research carried out in this paper can be considered as industrial (industry requested) application of machine learning in automated object detection. Object of interest—solder splash—is characterized by its small area and similar properties (texture, color) as its surroundings. The aim of our research was to apply state-of-the art algorithms based on deep neural networks for detection such objects in relatively complex electronic board. The research compared seven different object detection models based on you-look-only-once (YOLO) and faster region based convolutional neural network architectures. Results show that our custom trained YOLOv8n detection model with 1.9 million parameters can detect solder splashes with low detection speed 90 ms and 96.6% mean average precision. Based on these results, the use of deep neural networks can be useful for early detection of solder splashes and potentially lead to higher productivity and cost savings.

## Introduction

Currently, one of the fundamental ways to connect circuit components is through soldering. The baseplate and individual components must be perfectly connected when soldering power modules. Reduced costs in the manufacturing and assembly of the modules on the one hand, and ever-increasing demands on the module properties on the other, are what drive the constant development of the construction and connection technology of power modules^1^.

**Figure 1:**
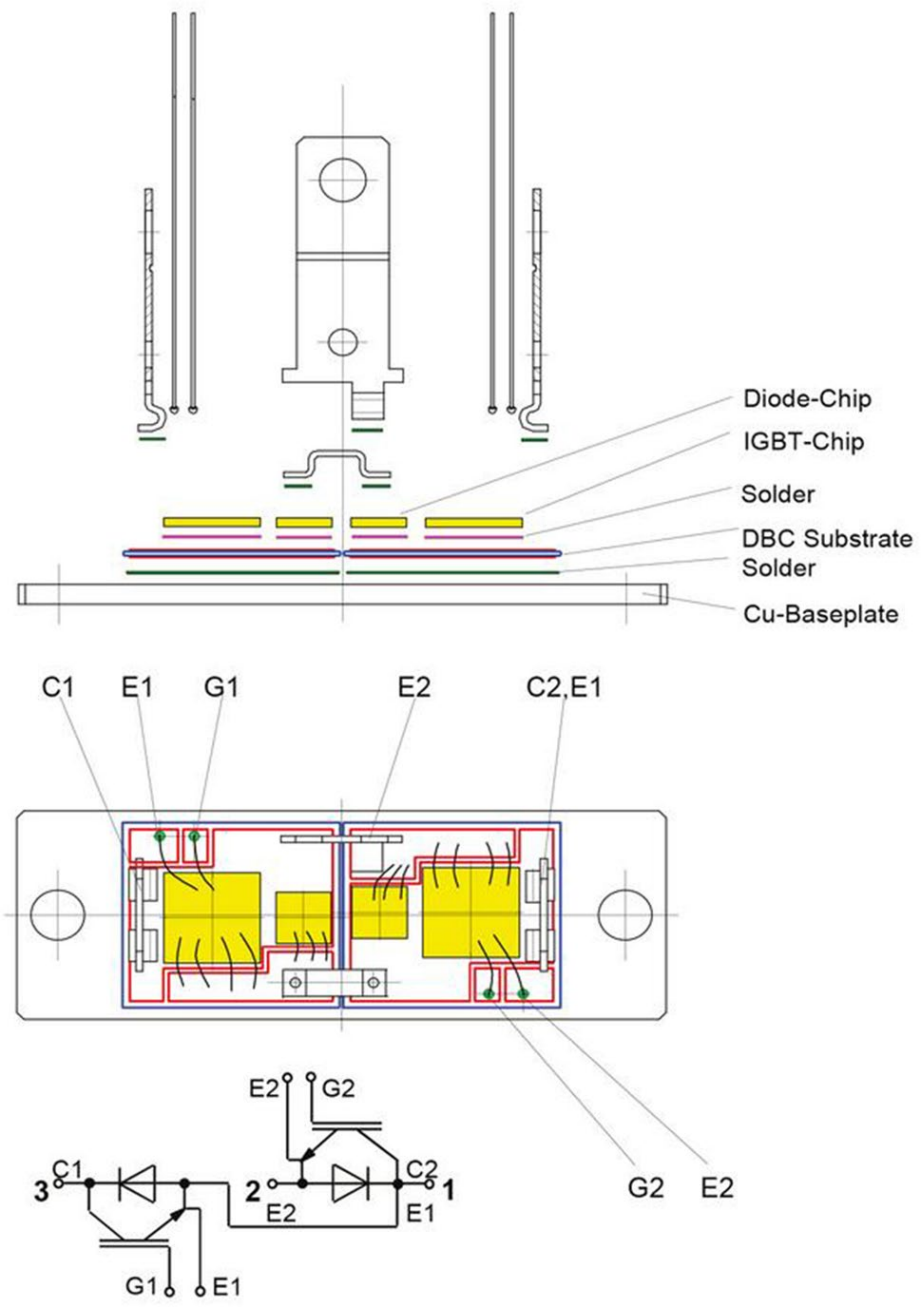
Basic concept of soldered power module SEMITRANS2^1^.

Materials and technologies used in the production process consist of various important steps. Multiple of them is focused on creating connections between different materials and layers with respect to increasing heat dissipation, and tighter and more flexible connections (Fig. 1).

If the soldering process is used during power module production, there are various problems, which can occur during the production of these modules. The power module can be soldered by:

- The most popular type of solder used in modern electronics assembly is solder paste. Solder paste is required for a surface mount application in order to secure the components to the circuit board. Solder paste might not be the only option, though. This is especially true when working with large devices or through-hole components that need more solder than printed solder paste can provide. In fact, a printed circuit boards (PCB) frequently uses hybrid technology and calls for more than one type of solder.
- Soldering material in exceedingly uniform shapes is known as a solder preform. The amount of solder delivered to the joint by each preform is consistent. To meet particular needs, solder preforms can be shaped into a range of sizes and shapes. The more popular shapes include frames, rectangles, squares, and discs. Depending on the amount of solder required to create the junction, sizes might range from very little to quite large. Tight tolerances can be maintained on the preform forms in critical applications, but if they are not required, they should not be specified because they raise the price of the solder preform.

**Figure 2:**
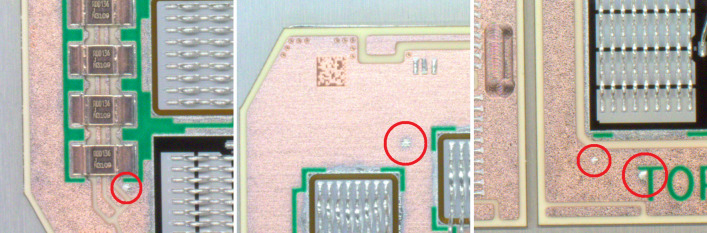
Typical examples of solder splashes in the power module manufacturing.

In praxis, it occasionally happens that components are not connected properly or that splashes are present while soldering individual elements (Fig. 2). The solder splashes very often occur when the soldering is applied to connect power modules. The first involves soldering silicon chips with solder paste, and then attaching power hybrid preforms to the baseplate and terminals.

The solder under the chips is melted during the soldering (or re-soldering) of power hybrids or module. From the solder, flux is leaking out to the chip edges. Solder splashes may result from a barrier or resistance to the flux’s migration, which can raise internal mechanical stresses. In production, there are restricted areas directly defined in product drawings where solder splashes cannot be found. If the solder splashes are in restricted areas, there is necessary to clean these areas.

The removal of the majority of identified solder splashes allows expensive electronic boards to be reused, which is significant from an economic point of view. Eliminating these defective areas within the module also improves the quality and reliability of the power electronic modules.

Optical contactless methods can be used for inspecting the power board modules and seem to be the crucial step in manufacturing process of electronic systems^2^. The soldering process of modules can be recorded by a high-resolution visual system. Capturing the area of interest with a high-resolution camera seems to be a good way for the post-processing of the manufacturing process. Manual inspection of PCB is still often used to detect the presence of solder splashes. With the development of image processing methods and machine learning, the automation of these processes through visual inspection is also coming to the fore^3^. The trend is to use convolution neural networks to detect circuit board defects^4–7^.

## Materials and methods

### Related work

The field of deep neural networks has gained popularity with the rise of available processing power, and storage space. Instead of using traditional feedforward neural networks, AI engineers have been gradually switching to deep learning models. The convolutional neural networks have a prominent place in the field of deep neural networks^8^.

Most actual works^9,10^ mentioned YOLOv8 as state-of-the-art methods for automated visual inspection of PCBs. Xiong shown that YOLOv8 has excellent accuracy 97% and Glučina et al. shown that smaller models (nano and small YOLO versions) are sufficient for this task.

Adibhatla et al. proposed deep learning model based on Tiny-YOLOv2 architecture for PCB quality inspection. Their dataset consisted of 11,000 images with image resolution 420 × 420 pixels. The images were prepared with help of the automated optical inspection (AOI) machine. The neural network model with approximately 15 million parameters achieved 98% accuracy for batch size 32. According authors, the YOLO architecture can be preferred due to detection speed and satisfactory accuracy. In comparison with Fast R-CNN network model, the YOLO model can achieve lover recall score and more localization errors^11^. The authors consider image subtraction and template-matching as traditional algorithms for PCB defect detection^12–14^.

Liao used modified YOLOv4 neural network model for detection of various PCB surface defects – broken line, hole loss, scratch, line repair damage, clutter and over oil-filling. Their dataset consisted of 2008 PCB defect images with image resolution 12-megapixel. The data augmentation increased the number of images to 19,029. Due to hardware limitations, the original images were resized to 416 × 416 pixels and labelled with annotation program LabelImg. The YOLOv4 model with 39.5 million of weight parameters achieved considerable performance with 98.6% mean average precision score (mAP)^15^.

Hu et al. used Faster RCNN neural network model with Feature Pyramid Networks for detection of small defects on the PCB. They classify 6 types of PCB defects – solder ball, open circuit, short course, mouse bite, spur and pinhole. Their detection model was tested in new PCB defects images and showed valid results. Authors further consider their model especially suitable for use in (PCB) production environment.

“Deep neural networks are more suitable for large targets detection”^16^. ”Object detection using high-resolution pictures can be slow for purposes of real-time detection”^16^.

According Hu et al. the improving of detection accuracy is often in focus instead of real-time detection for production purposes^16^.

Su et al. compared 3 types of neural networks for semiconductor wafer post-sawing inspection: radial basis function network, feedforward with backpropagation and learning vector quantization. Their approach could shorten the object detection time to 1 s per slice^17^.

Since the automated PCB defects inspection often relies on image processing, the convolutional neural networks became successful application of machine learning algorithms. The YOLO (You look only once) and Faster R-CNN models belong to the most used computer algorithms in the field of automated objects detection^8,16,18^.

**Figure 3:**
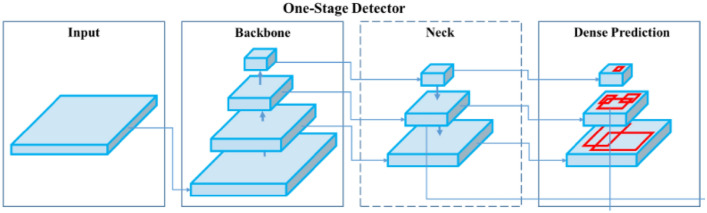
The data flow of one-stage object detector model.

The YOLO model is considered as state-of-the-art object detection system. The detection speed and satisfactory accuracy are main advantages of this model. With a sufficiently powerful graphics card, the detection system can be considered as real-time. The YOLO algorithm is one-stage detection system (Fig. 3) consisting of the backbone part, the neck part and the head part. The input data (images, patches, …) are fed to backbone part. The backbone is a pre-trained network model for extraction of useful image features. The ResNet-50, Darknet53 and VGG16 are among the most used ones. Further, the neck part extracts “feature pyramids” in order to better generalize on various image scales and resolutions. Finally, the head part performs final stage operations as anchor boxes and renders final outputs: class name, bounding boxes and confidence score^19,20^.

Since 2016, eight versions of this algorithm have been released. We focus on YOLOv5 and YOLOv8 versions released by Ultralytics in 2015 and 2022 respectively. The first one is widely used in area of automated object detection and the second one is the last version with new features and improvements^20^.

YOLOv5 has achieved state of-the-art performance in terms of classification accuracy on the COCO dataset, with average precision 50.5%. It is well optimized for real-time applications with remarkable FPS (frame per second). In comparison with other YOLO versions, the YOLOv5n achieved highest FPS score. The detection of small objects can be problematic, however YOLOv5 as well as YOLOv8 has shown excellent performance in this area (topic). The last version of YOLO model has outperformed YOLOv5s in terms of accuracy score (54.2% vs. 51.4%). Both YOLOv5 and YOLOv8 models use CSP Darknet53 backbone architecture, anchor boxes, same optimizer, post-processing and non-maximum suppression (NMS)^18,20^. We have decided to use Ultralytics software implementation of YOLO algorithm. The documentation, large community and high detection speed were the main reasons.

**Figure 4:**
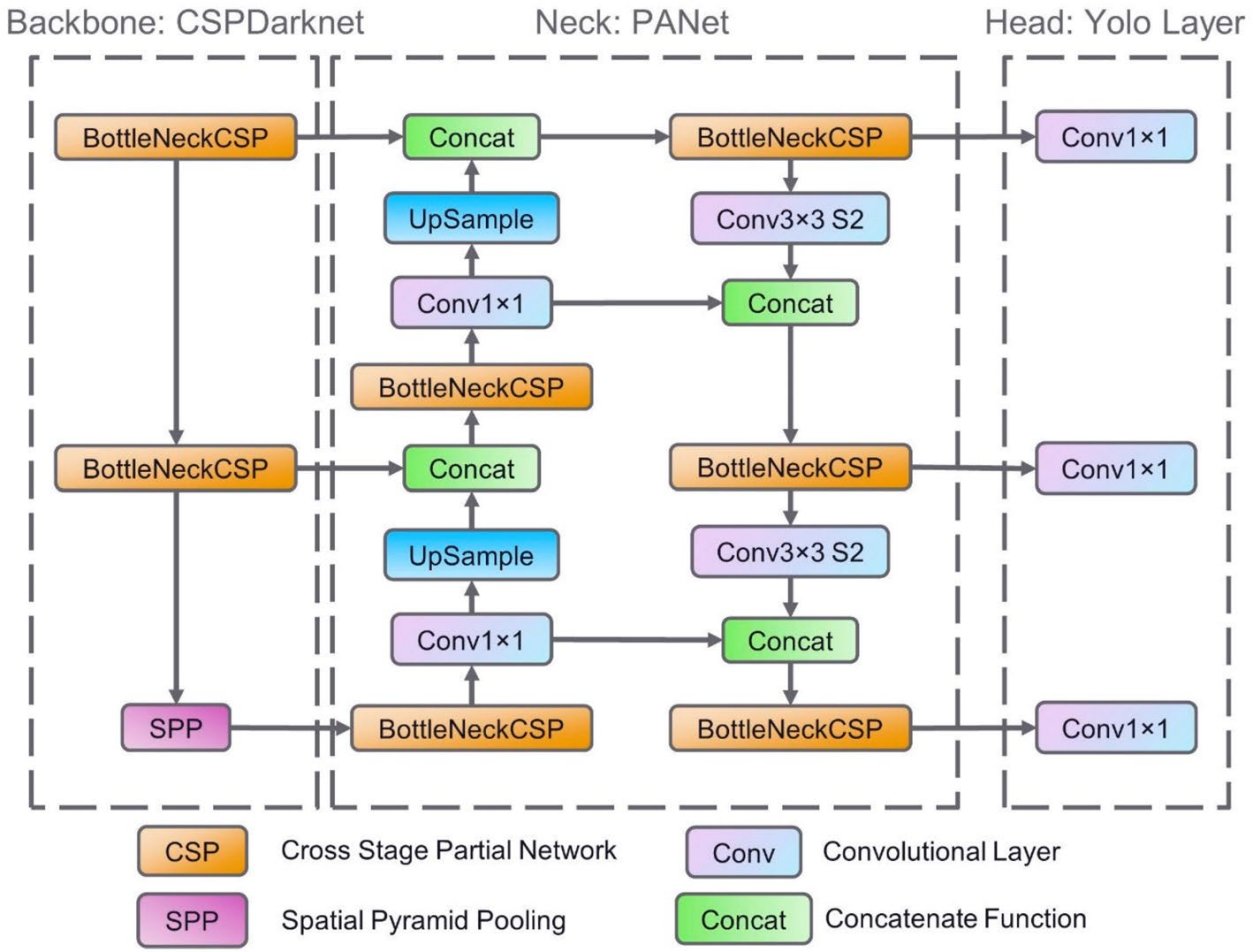
The neural network architecture of YOLOv5.

The Fig. 4 shows the architecture of YOLOv5 model. It consists of three blocks: (1) Backbone CSP-Darknet53, (2) Neck: PANet and last block (3) Head: YOLO layer. The input data are first fed into feature extraction layer. Then these data are processed in the PANet block and finally the last block give output detection parameters: predicted class, confidence score, location and size.

**Figure 5:**
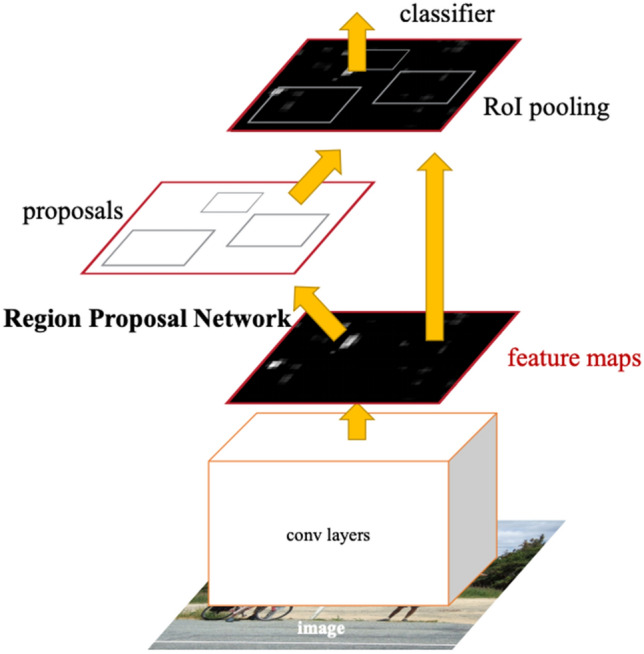
The Faster R-CNN architecture ^21^.

Faster R-CNN architecture belongs to the most used algorithms for automated object detection and has achieved excellent results on most benchmark data sets (COCO, ImageNet, MNIST)^21^. The Faster R-CNN detection model is composed of two blocks. The first block (Region Proposal Network) is deep convolutional network with main task to propose image regions. The second block is the Fast R-CNN detector module that further process proposed regions. These two blocks form single network for object detection (Fig. 5). We can look at the Region proposal network (RPN) as ‘attention’ module which tells the Fast R-CNN block where to look^18,21^.

This architecture originates from previous versions R-CNN and Fast R-CNN, however, has greater accuracy score and its region proposal part is deep neural network. Unlike previous versions, Faster R-CNN use the same feature map for region proposals and objects classification. The Faster R-CNN architecture contains backbone network for extraction of image features. The ResNet network combined with Feature Pyramide Network are often used for this task. Further, the anchor generation process follows that outputs anchor boxes with variable aspect ratios and resolutions^22^.

The primary task of RPN module is detection of anchor boxes, those will achieve in next layer best classification results. The RPN module is composed of regression and classification part. The first one predicts potential object in the anchor box and the second one predicts space variations of objects in anchor boxes with ground truth labels to fit them as precisely as possible^21,23^.

### Searching of model parameters (Adam algorithm)

The optimization based on gradient computation belongs to core tasks in data science and engineering. The minimization of differentiable error function with respect to weight coefficients is key part of the neural networks supervised learning. The main advantage of gradient descent algorithm is relatively small computational complexity of first-order partial derivatives of error function w. r. t. weight parameters^8^.

The Adam algorithm combines the advantages of popular AdaGrad and RMSProp optimization methods. The magnitudes of weight updates are invariant to gradient rescaling and the steps are approximately bounded by the stepsize parameter. The Adam algorithm works also with sparse gradients and its memory requirements are relatively small^24^.

Let´s have the system of equations:

1 $${g}_{t}={\nabla }_{\theta }{f}_{t}\left({\theta }_{t-1}\right)$$

2 $${m}_{t}={\beta }_{1}\cdot {m}_{t-1}+\left(1-{\beta }_{1}\right)\cdot {g}_{t}$$

3 $${v}_{t}={\beta }_{2}\cdot {v}_{t-1}+\left(1-{\beta }_{2}\right)\cdot {g}_{t}^{2}$$

4 $${\widehat{m}}_{t}={m}_{t}/\left(1-{\beta }_{1}^{t}\right)$$

5 $${\widehat{v}}_{t}={v}_{t}/\left(1-{\beta }_{2}^{t}\right)$$

6 $${\theta }_{t}={\theta }_{t-1}-\alpha \cdot {\widehat{m}}_{t}/\left(\sqrt{{\widehat{v}}_{t}}+\epsilon \right)$$

Let *f(θ)* be a scalar error function: that is differentiable w.r.t. parameters *θ* (weight coefficients). The aim of optimization is the minimization the expected value of this function, *E[f(θ)]* w.r.t. its parameters *θ*. We denote the timesteps of stochastic error function as *f1(θ), …, fT (θ)* and subsequent timesteps 1, …, T. The Eq. (1) describes the gradient w.r.t *θ* at timestep *t* as *g*_*t*_ = *∇*_*θ*_* f*_*t*_* (θ)*. The learning step size denoted as *α* is often set to 0.001 and parameter *ϵ* is set to 10^–8^. The hyperparameters *β*_*1*_*, β*_*2*_ indicates exponential decay rates for moment estimates and the most of software implementations set them to 0.9 and 0.999 respectively. The neural network weight vector is denoted as *θ*_*0*_. The 1st and 2nd moment vector are denoted as *m*_*0*_ and *ν*_*0*_ respectively and initialized to 0. The Eqs. (2) and (3) represents algorithm updates of exponential moving averages of the gradient *m*_*t*_ and the squared gradient *v*_*t*_ respectively. The Eqs. (4) and (5) denotes the computation of the 1st moment and the 2nd raw moment (the uncentered variance) estimates of the gradient. The update of weight parameter vector according Adam algorithm is expressed in Eq. (6)^24^.

### PCB image acquisition

**Figure 6:**
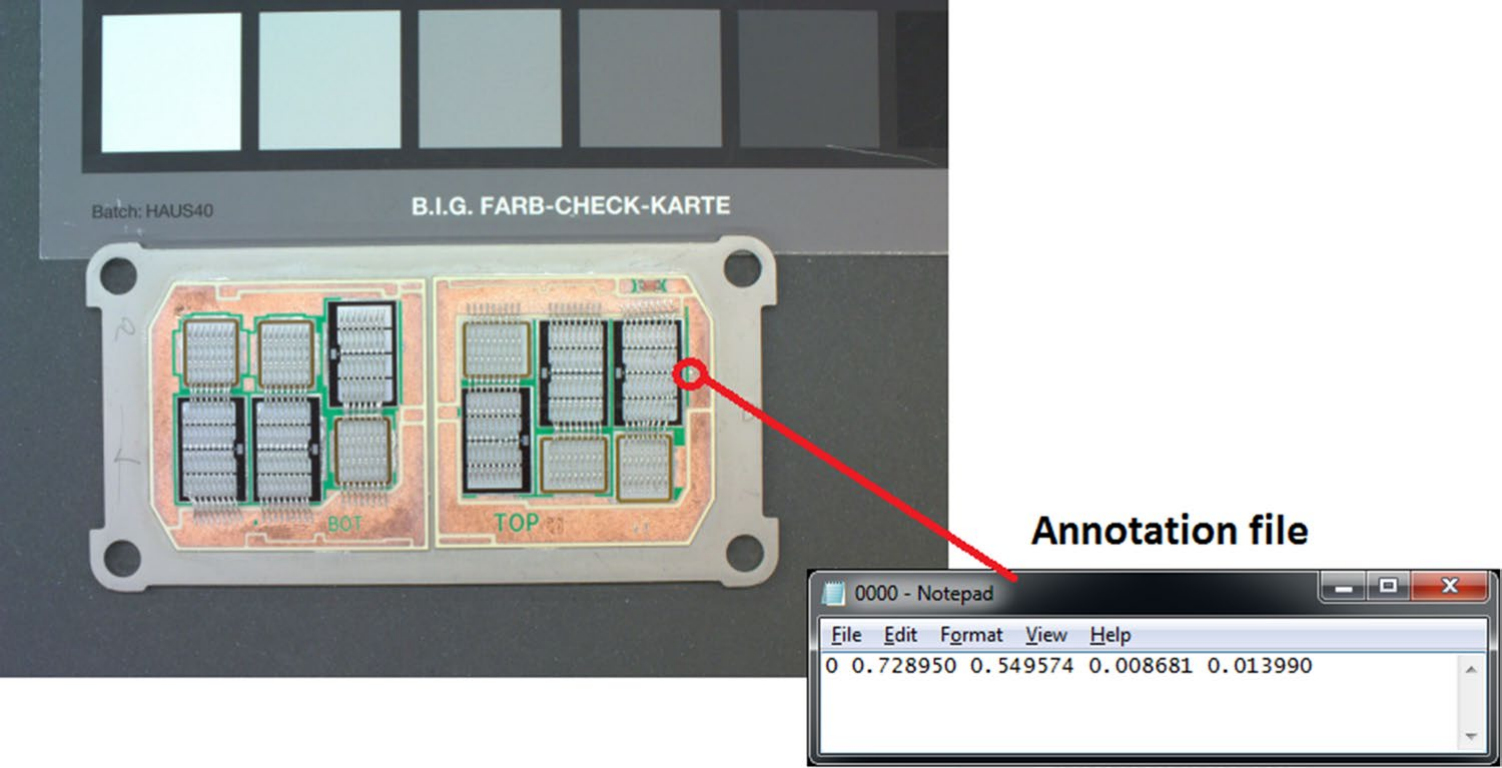
PCB image in dataset with related annotation.

For training the neural network we created our own dataset of PCBs under the test in given manufacturer. Respecting the fact, that object of interest – solder splash – is small in comparison to size of entire PCB, we used color USB camera with high resolution (15 MPix Basler acA4600-10uc). The PCBs images were captured in real manufacturing environment (preserving mainly lighting conditions and color parameters). In the first experiment we captured approximately 200 PCBs containing at least one solder splash. The images were annotated by quality specialists of manufacturer using LabelImg software. The annotation text file uses YOLO annotation format. In this text file each object is descripted in the separate line by number of class; relative position of bounding box center and relative width and height of object bounding box. Only one class of objects was created: 0 – solder blob. The example of PCB image with annotation text file is shown in Fig. 6.

### Tiling

When the resolution of input images is too high (in our case the resolution is 4608 × 3288 pixels), YOLO training process is GPU memory consuming. Due to this fact we divided these images into a series of subimages (tiles) with constant size (640 × 640 pixels). In our pilot study^25^ we tiled the original images from the origin (left-top pixel) regardless objects of interest. In many cases the objects of interest were intersected by tile boundaries and the object was not detected properly (we had several false negative cases) and algorithm performance was decreased.

**Figure 7:**
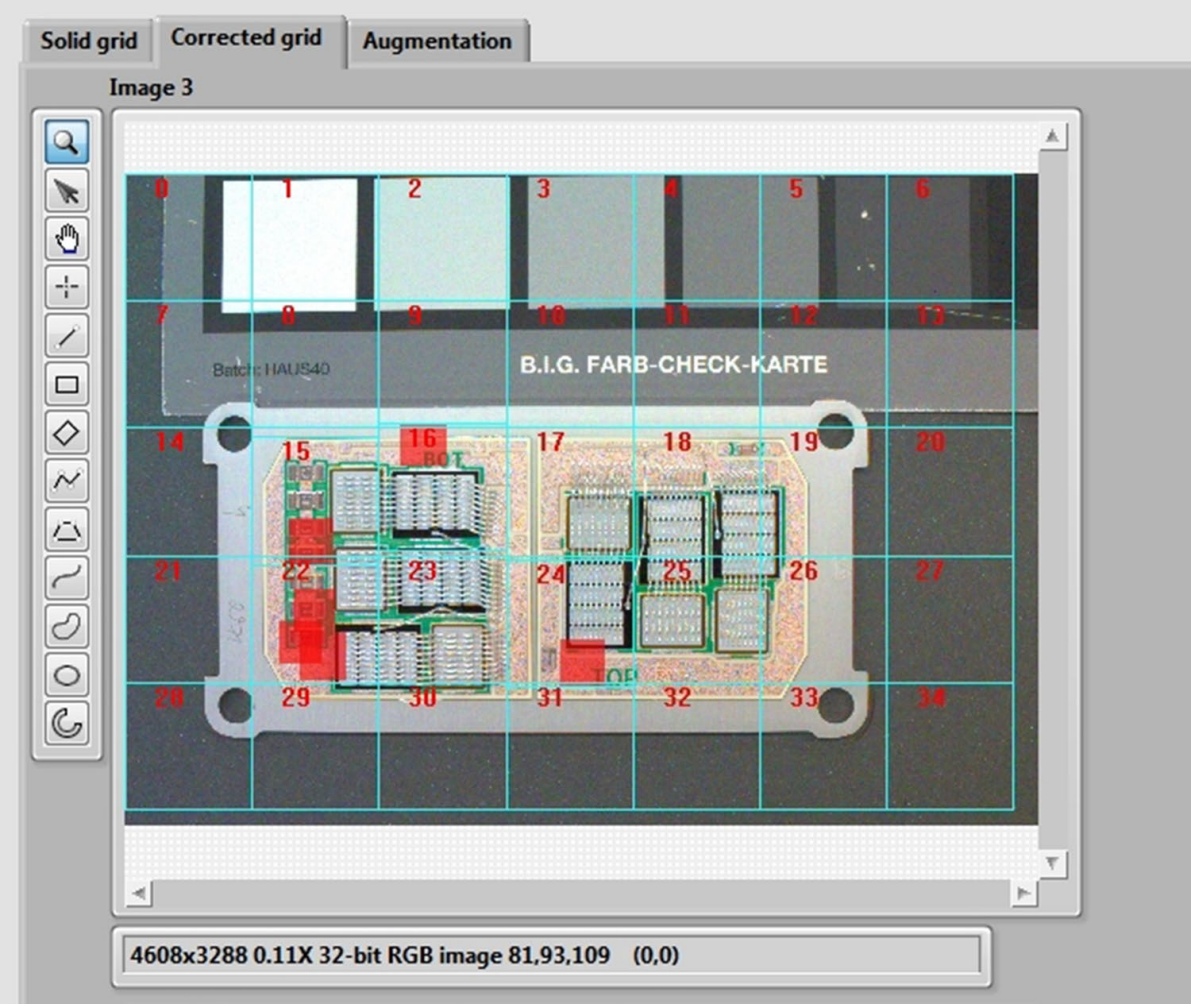
Image tiling implemented into the LabVIEW environment – front panel detail.

**Figure 8:**
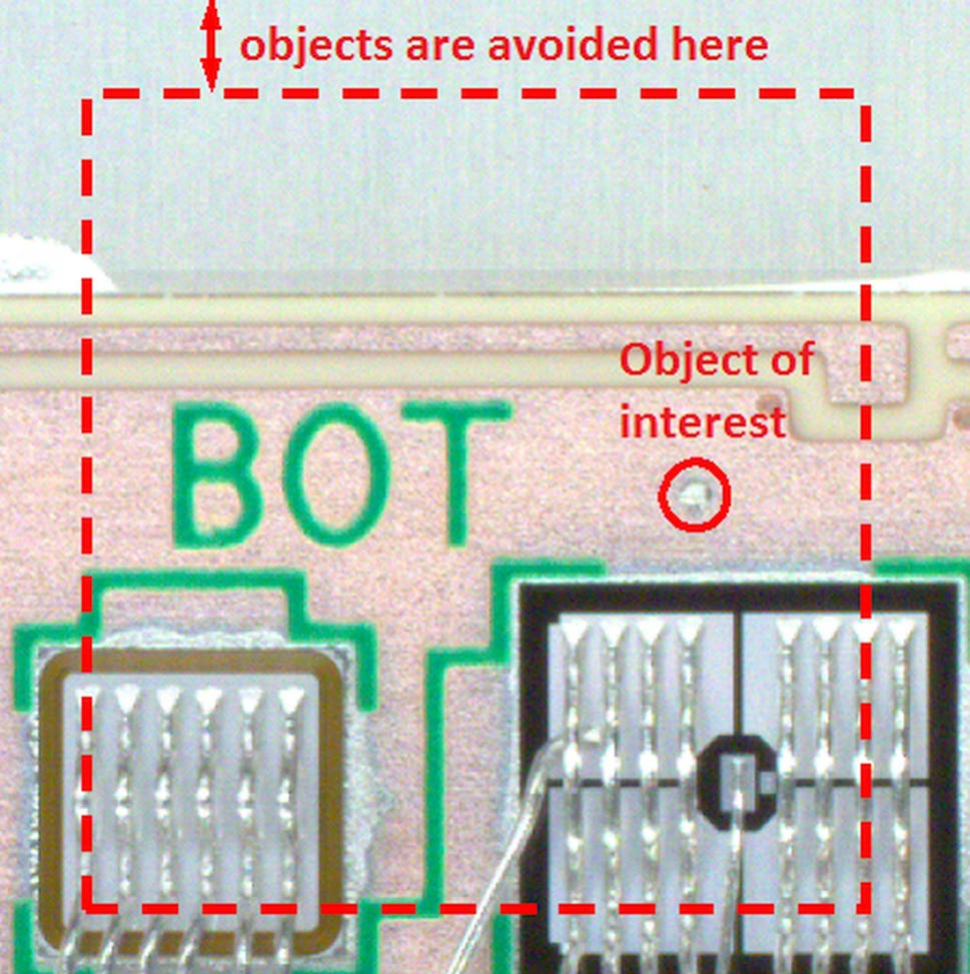
Proper output of tiling algorithm – tile 640 × 640 pixels with 1 object of interest.

For this reason, we developed new tiling algorithm respecting the objects of interest positions, where the boundaries of each tile must not intersect the objects of interest. Tiles can overlay each other. We also applied the condition that object of interest must have at least 50-pixel distance from each boundary to secure optimal location in detection process. In the Fig. 7 we can see the tiling process implemented in LabVIEW development system with proposals of tiles in blue boxes. Objects of interest are centered under the red boxes (boxes are extended by desired minimal distance of object of interest from the tile boundaries). The tiling algorithm starts with solid grid 640 × 640 pixels from the image start position (left-top corner). If any intersection is detected, new position of given tile is computed based on position parameters of all objects from original annotation file for full resolution image. Also manual shift of each tile is enabled in this algorithm. Tiling algorithm need to recalculate the annotation text file for each object in given tile according to YOLO annotation format. Proper output tile from our tiling algorithm is shown in Fig. 8.

### Dataset augmentation

Our pilot dataset contains 198 images with full resolution of 15 MPix. As known in this field of computer vision, to improve detection algorithm accuracy and to extend the initial dataset some augmentation can be provided. Data augmentation is a technique in machine learning used to reduce overfitting when training a machine learning model, by training models on several slightly-modified copies of existing data^26^. As augmentation techniques we decided use those modifications which can typically occur in real manufacturing environment: variations of light (homogeneity and temperature), blurring or noise occurrence.

In the Table 1, the augmentation techniques are briefly described. All the images were modified using NI LabVIEW environment and IMAQ functions.

**Table 1:** Description of augmentation methods.

Augmentation modification	Description
#1 – adding weak noise	Random number from range − 10 to + 10 with Gaussian distribution is added to all R, G and B channel if intensity of pixel in all channels is in range 50–200
#2 – adding stronger noise	Random number from range − 15 to + 15 with Gaussian distribution is added to all R, G and B channel if intensity of pixel in all channels is in range 50–200
#3 – increasing brightness linearly	The brightness of all R, G and B channel is linearly shifted from default value 128 (parameter of linear transfer function in lookup table)^27^ to the same random number from range 130–145
#4 – decreasing brightness linearly	The brightness of all R, G and B channel is linearly shifted from default value 128 (parameter of linear transfer function in lookup table)^27^ to the same random number from range 85–100
#5 – Gaussian blur	The image is blurred with convolution filter using standard Gaussian kernel and the size of kernel is randomly switched between 3 × 3 and 5 × 5
#6 – color balance	Random brightness increasing is applied to R or B channel to simulate the change of color temperature. For warmer light, the R channel is linearly increased with number from interval 130–150. In the case of colder light, the B channel is modified the same way

Influence of augmentation is shown in the Table 2.

**Table 2:** Influence of augmentation.


Augmentation 1	Augmentation 2	
Augmentation 3		Augmentation 4
Augmentation 5		Augmentation 6

At the end of augmentation process, our dataset was extended to 24 000 tiles with resolution 640 × 640 pixels. For the training process we used mostly those of them containing at least one solder splash (99%, 1 500 tiles), 1% were images containing only background (without solder splash)^20^.

## Experimental results

**Figure 9:**
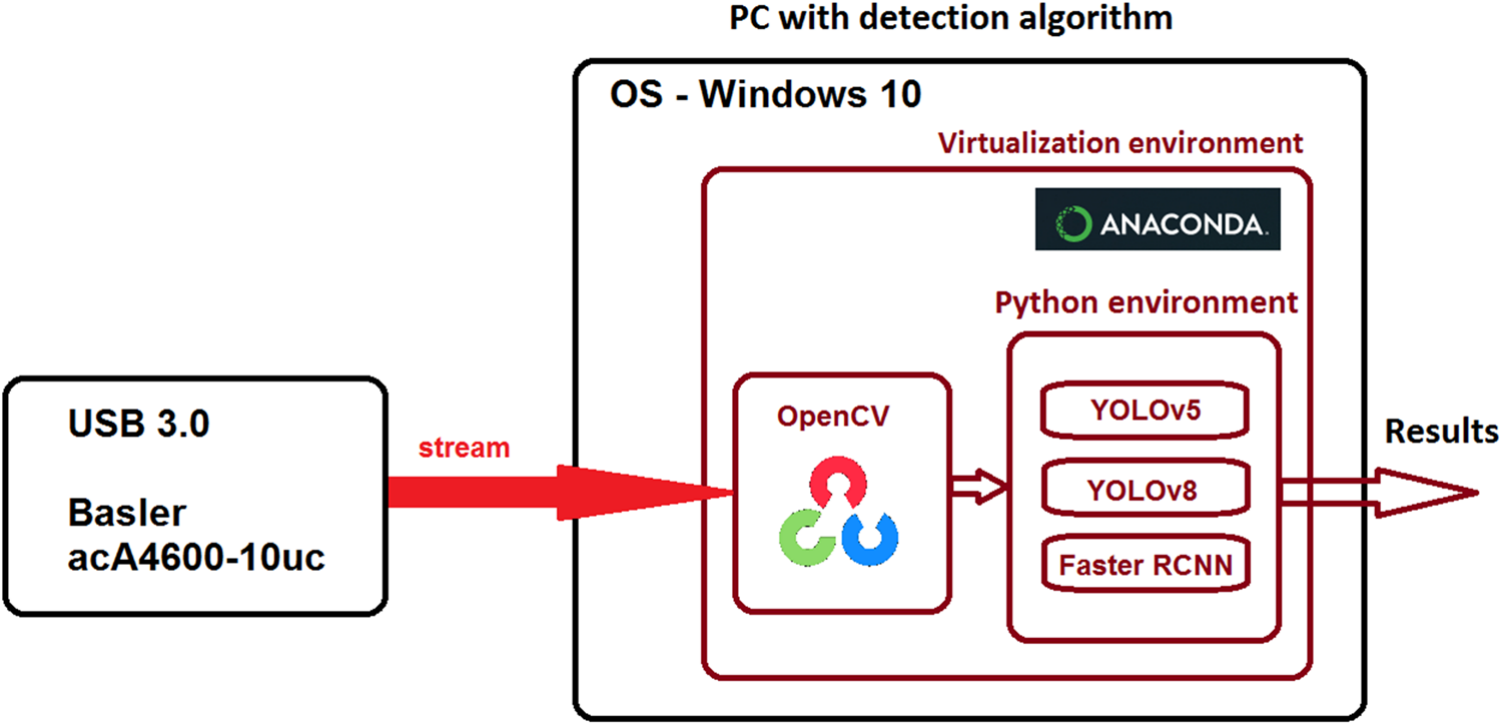
Block diagram of used software environments in practical implementation.

All software experiments were implemented in Python3.9. using a model developed based on PyTorch 1.13^28^, which provides a library for building an architecture for deep learning models. The experiment was performed on an AMD Ryzen 5600X CPU @ 3.7 GHz, NVIDIA GeForce RTX 3060 GPU, and 32 GB RAM on Windows 10 OS. The functional block diagram of software environments is depicted in Fig. 9.

In the task of automated detection of solder splashes we have compared seven different object detection algorithms. Five of them were classified as YOLO detection models and remaining two were Faster RCNN methods. The main properties of chosen computer models are shown in (Table 3). The computational complexity is expressed in floating point operations (Flops). It is important parameter of the detection algorithm related to computational requirements of hardware. The detection speed of model is another important factor in the task of automated inspection of PCBs. Considering these important parameters, the YOLOv8n model was showed as compromise between detection speed and detection accuracy. The mean average precision score (mAP) describes neural network model accuracy – the higher value indicates more accurate object detection model. The comparison for selected neural network models is shown in Table 4.

**Table 3:** Basic parameters of the neural network models. The values in bold represent the model chosen as trade-off between detection speed and detection performance.

Network	Parameters [10^6^]	Flops [10^9^]	Speed [ms]	mAP [%]	Epochs
YOLOv5n	1.9	4.5	60	0.93	500
YOLOv5s	7.2	16.5	110	0.937	500
YOLOv5m	21.2	49.0	230	0.946	500
YOLOv8n	**3.2**	**8.7**	**90**	**0.966**	**250**
YOLOv8s	11.2	28.6	145	0.940	250
Faster RCNN (ResNet50)	41.4	134	480	0.920	100
Faster RCNN (ResNet50-FPN)	43.7	280	690	0.950	100

Significance values are in [bold].

**Table 4:** Basic parameters of the neural network models. The values in bold represent the model chosen as trade-off between detection speed and detection performance.

Network	True positives	False negatives	False positive	Precision	Recall
YOLOv5n	279	20	21	0.93	0.933
YOLOv5s	281	18	19	0.937	0.940
YOLOv5m	280	19	16	0.946	0.936
YOLOv8n	**283**	**16**	**10**	**0.966**	**0.947**
YOLOv8s	286	13	17	0.940	0.957
Faster RCNN (ResNet50)	275	14	14	0.920	0.952
Faster RCNN (ResNet50-FPN)	284	11	9	0.950	0.963

Significance values are in [bold].

Every object detection model was launched 10 times with different pseudorandom number generator seed settings (PRNG). It is the PRNG that controls quasi-random division of data in train/validation/test set and various model parameters. Different seed value ensures stochasticity in learning process and potentially leads to global optima of gradient error function. Best result of 10 learning replicas for each neural network model is reported in Table 4. The true and false positive values in precision score (7) are related to: correctly detected solder splashes and image area incorrectly detected as solder splashes respectively.

7 $$Precision= \frac{True Positive}{True Positive+False Positive}$$

The recall score (8) also contains the false negatives parameter related to undetected solder splashes objects.

8 $$Recall= \frac{True Positive}{True Positive+False Negative}$$

**Figure 10:**
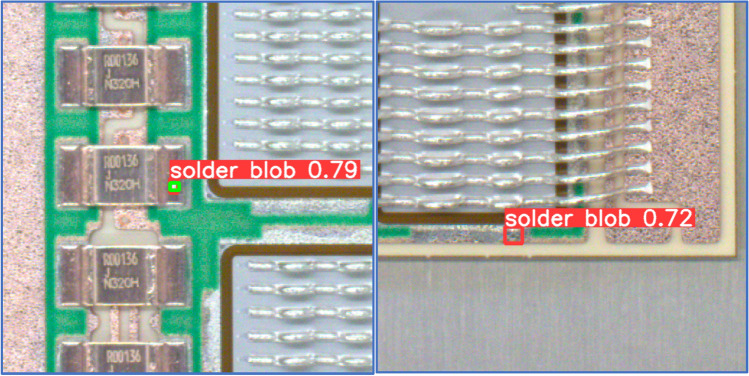
Left figure shows correctly detected small solder splash, right part shows object incorrectly detected as solder splash. True position of solder splash is marked with green rectangle; the algorithm’s result is marked with red color. The number denotes confidence of object detection.

As we can see on Fig. 10, the YOLOv8n detection algorithm can localize even solder splashes with small area. Despite the fact, that the PCB pictures contained many objects similar in texture, color and shape to solder splashes, the number of false positives were below 4%.

**Figure 11:**
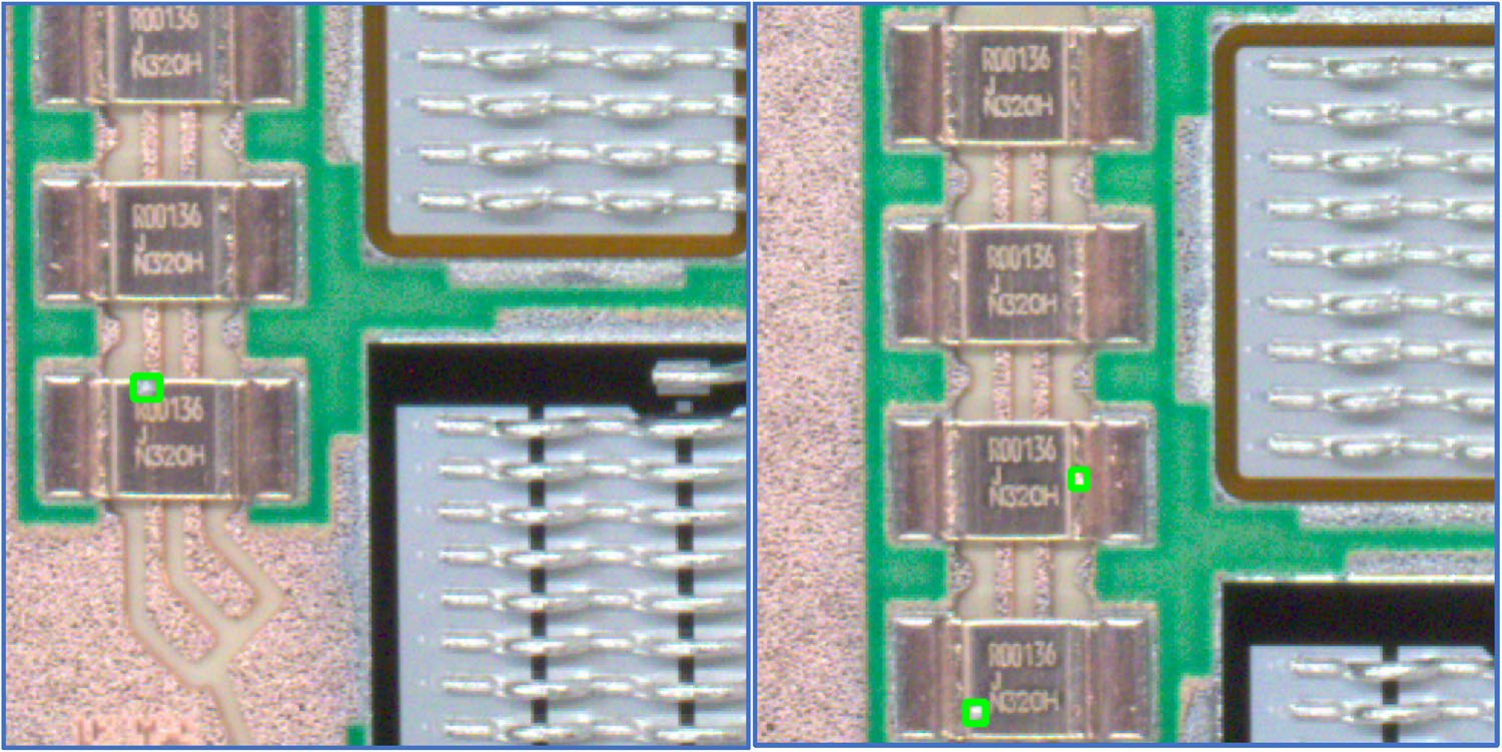
The figures show undetected small solder splashes (false negatives). True position of solder splash is marked with green rectangle.

The Fig. 10 contains the YOLOv8n models undetected solder splashes. We can see on Fig. 11 the YOLOv8n detection algorithm can localize even solder splashes with small area. The number of false negatives were in case of YOLOv8n model below 5%.

From one-stage object detection methods the YOLOv8s reached highest recall score and YOLOv8n reached highest precision score. Due to computational complexity and processing speed, the large and extra-large versions of YOLOv5 and YOLOv8 models were not considered.

The Faster RCNN two-stage model with ResNet50-FPN backbone slightly outperformed the YOLOv8s in the Recall score (0.963 vs. 0.957).

The Ultralytics implementation of YOLOv5 and YOLOv8 object detection algorithms includes configuration file with dozens of learning hyperparameters. In order to improve object detection performance, several hyperparameters were repeatedly changed. The best results were obtained with parameters settings (Table 5). The default learning rate was at least 10 times lowered. Authors in^20^ recommended the amount of background images (no solder splashes) only 1–10% of all images. We confirm this statement—the best results were obtained with 2–3% background images of the total set.

**Table 5:** YOLOv5 and YOLOv8 learning hyperparameters. These parameters provided best detection performance (Ultralytics implementation, Pytorch framework).

Initial learning rate	0.0001
Optimizer algorithm	Adam
Batch	16
Number of epochs	1000
Image HSV value augmentation	0.0
Patience	300
Image resolution	640

**Figure 12:**
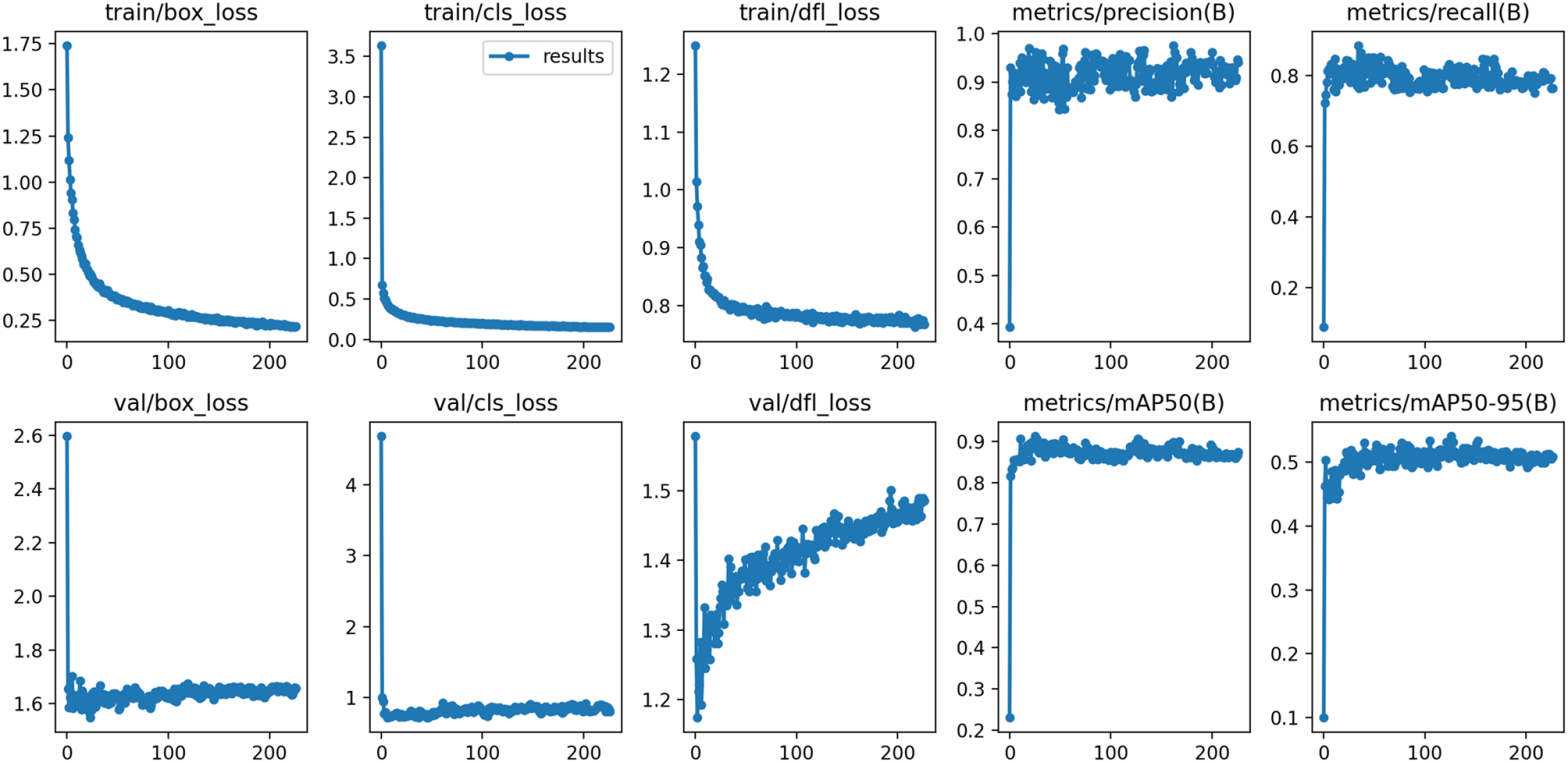
The YOLOv8n model learning curves for training (train) and validation (val) set. The x-axis denotes the training iteration (epoch).

As we can see on the Fig. 12, the relevant metrics measuring YOLOv8n model detection performance showed decreasing trend over the epochs. The box loss (box_loss) parameter represents the algorithm’s accuracy of object’s center localization. The objectness loss (obj_loss) represents the object’s probability of detection in a proposed region of interest (ROI). Both parameters were decreased in training and validation set as well. These parameters showed good generalization potential of YOLOv8 model in localization of soldering splashes centers. The training phase of YOLOv8 detection model was early stopped after the increase of objectness loss in validation set. The trained model showed very good solder splashes detection capabilities.

**Figure 13:**
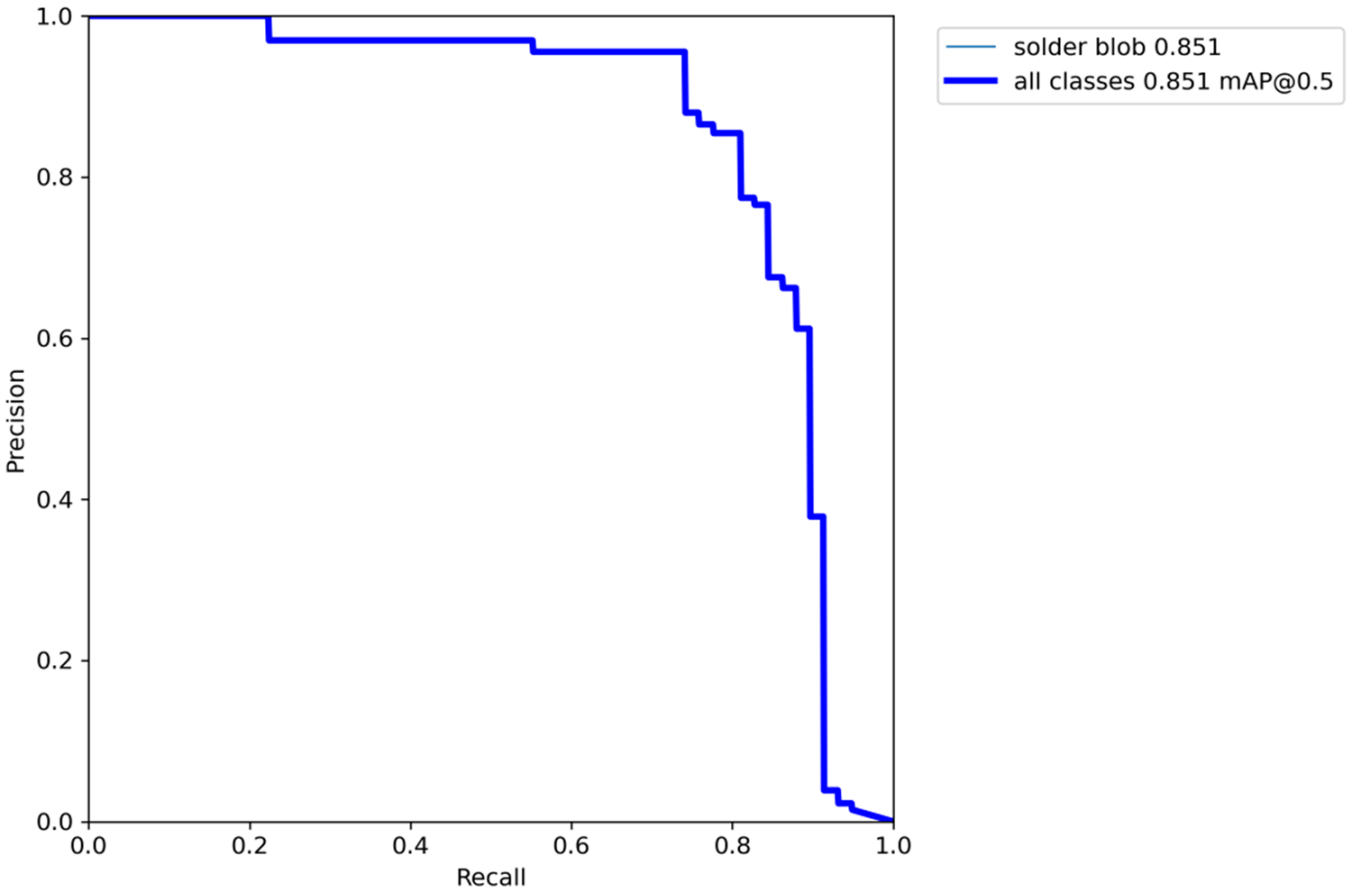
The YOLOv8n model learning curves for training (train) and validation (val) set. The x-axis denotes the training iteration (epoch).

The Precision score dependence from recall score under different confidence thresholds is shown in the precision-recall curve (Fig. 13).

## Discussion

In this section we discuss the results from multiple points of view. The aim of our research was detection and localization of single class PCB defect – solder splash. Our results show that our custom trained YOLOv8n algorithm is able to detect and localize the solder splashes in images of specific power electronics PCB boards with considerable precision.

From a machine learning perspective, the automated detection of solder splashes in our application, was a real challenge. The main reason was the fact, that the PCB pictures contained many objects similar in texture, color and shape. The small area of solder splash object was another reason.

Considering related work mentioned in section “Related work” and industry-oriented focus of our research, we have decided to compare only the most used and most successful object detection methods: Faster RCNN and YOLO. Both models belong to the state-of-the-art methods for automated object detection and classification. Seven different versions of detection algorithms were tested: three YOLOv5, two YOLOv8 and two Faster- RCNN versions.

Despite the fact there are many scientific articles related to automated solder splashes detection, our research is original in some aspects.

Solder splashes in our PCBs are very small related to the PCB dimensions. This is the main reason why input images have the high resolution (15 MPix). Authors in many references used images with smaller spatial resolution or object of interest was bigger related to the image area.

Splashes are surrounded by background with similar textural or structural properties (background differs from splashes often in color variations, wire bonds with light reflections – see detailed images in Table 2 as example). These facts can cause complications with objects of interest detection.

Application of deep neural networks models for such small objects and other complications mentioned above was real challenge. YOLOv8 model was selected as state-of-the-art method and also for compromise between model accuracy and detection speed (requested in serial manufacturing).

The YOLOv8n model has achieved the highest detection performance 96.6% mAP and second lowest detection speed 90 ms with 3.2 M network parameters. The YOLOv5n model has achieved the best detection speed – only 60 ms, but at the cost of lower value of mAP (93%). The low detection speed is mainly caused by the relatively small number of model parameters (1.9 M). In comparison with five different YOLO detection models, the Faster-RCNN method with ResNet50-FPN backbone has achieved the highest recall score 96.3% and second highest mAP 95%. Hovewer, in comparison with the YOLOv8n model, its detection speed was at least 7 times higher (690 ms vs. 90 ms). The Faster-RCNN method has also much higher data storage requirements (41.4 M parameters vs. 3.2 M parameters).

Considering the detection speed, data storage requirements and detection performance, we prefer the YOLOv8n model for automated solder splashes detection.

## Conclusions

In the presented work, we propose application of machine learning object detection algorithm in the area of PCB defects detection. We have only considered one category of PCB defect – solder splash. The presented results have strong assumption that the training and testing data are sampled under the same lighting conditions from the same dataset. Since the aim of our research was not to detect solder splashes in all kinds of PCB’s, the presented application of well-known YOLO detection method turned out to be a satisfactory solution for automated detection.

Presented YOLOv8n detection model with 1.9 M parameters is lightweight, has low detection speed 90 ms and excellent mAP 96.6%.

Based on these results, the integration of deep neural networks in the process of automated optical inspection (AOI) can be useful for early detection of PCB defects and potentially lead to higher productivity and cost savings.

If there are some slight variations of PCB size, topology, lightning and color conditions, this has negligible effect on detection accuracy. Very important is to preserve the lightning and color conditions in images. The Semikron-Danfoss company knows that fact and they maintain normalized lighting conditions. If the change of mentioned parameters is more significant, dataset and training process should be updated.

The enlargement of the dataset size and increasing the spatial resolution of PCB images can be object of future research and potentially lead to higher detection score. Another subject of future research can be detection of other categories of PCB defects.

## Data Availability

The images and annotations files of the training and validation set are available in the Solder_splashes repository, https://github.com/Peet62/Solder_splashes.
